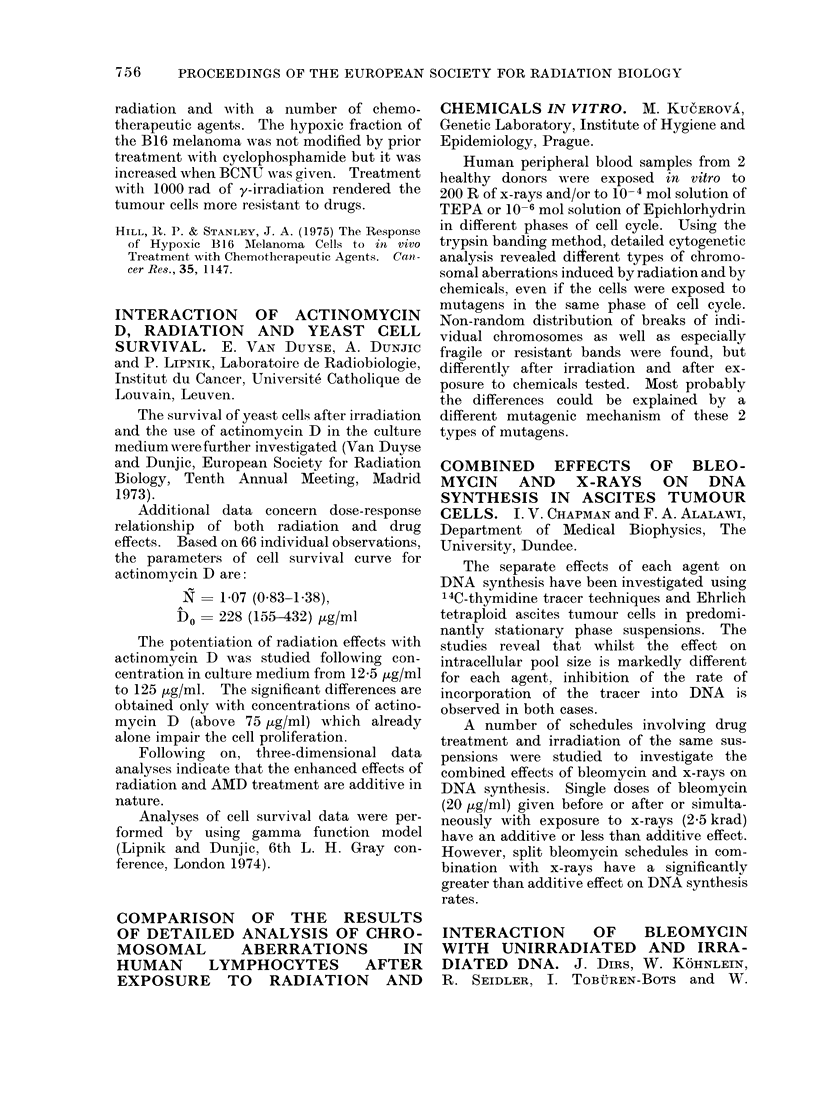# Proceedings: Combined effects of bleomycin and x-rays on DNA synthesis in ascites tumour cells.

**DOI:** 10.1038/bjc.1975.306

**Published:** 1975-12

**Authors:** I. V. Chapman, F. A. Alalawi


### COMBINED EFFECTS OF BLEOMYCIN AND X-RAYS ON DNA SYNTHESIS IN ASCITES TUMOUR CELLS.

I. V. CHAPMAN and F. A. ALALAWI, Department of Medical Biophysics, The University, Dundee.

The separate effects of each agent on DNA synthesis have been investigated using $^{14}C$-thymidine tracer techniques and Ehrlich tetraploid ascites tumour cells in predominantly stationary phase suspensions. The studies reveal that whilst the effect on intracellular pool size is markedly different for each agent, inhibition of the rate of incorporation of the tracer into DNA is observed in both cases.

A number of schedules involving drug treatment and irradiation of the same suspensions were studied to investigate the combined effects of bleomycin and x-rays on DNA synthesis. Single doses of bleomycin (20 $\mu$g/ml) given before or after or simultaneously with exposure to x-rays (2·5 krad) have an additive or less than additive effect. However, split bleomycin schedules in combination with x-rays have a significantly greater than additive effect on DNA synthesis rates.